# Supplementary material for: Economic Evaluation of Nemonoxacin, Moxifloxacin and Levofloxacin in the Treatment of Early Community-Acquired Pneumonia with Possible Pulmonary Tuberculosis
Source: Int J Environ Res Public Health. 2022 Apr 15;19(8):4816. doi: 10.3390/ijerph19084816 (PMC9028707; doi:10.3390/ijerph19084816)
Supplement: Supplementary file 1 [file ijerph-19-04816-s001.zip › ijerph-1656661-supplementary.pdf]

Supplementary Materials

- Table S1 Basic information of clinical research (pages 2)
- Table S2 Dosing regimen (pages 2)

**Table S1 Basic information of clinical research**

| Group<br>drug dosing(number of patients) | Phase II nemonoxacin 500mg (63) | Phase II moxifloxacin 500mg (60) | Phase III nemonoxacin 500mg (312) | Phase III levofloxacin 500mg (166) |
|---|---|---|---|---|
| Age (mean and standard deviation) | 52.7(15.33) | 51.6(16.00) | 57.8(14.18) | 56.5(14.62) |
| Male (proportion of males,%) | 62.7 | 64.1 | 62.1 | 62.7 |
| Ethnicity (Proportion of Han nationality,%) | 92.5 | 95.3 | 96.8 | 97.6 |
| Height (cm) | 163.5(7.08) | 164.7(8.68) | 164.9 (7.57) | 165.5 (6.88) |
| Weight (kg) | 60.0(8.45) | 64.3(11.81) | 62.4 (10.60) | 62.7 (10.69) |
| BMI(kg/m2) | 22.5(3.01) | 23.6(3.29) | 22.9 (3.18) | 22.8 (3.23) |
| Pneumonia Severity Index score | Level 2: 46.3%,Level 3: 50.7%,Level 4: 3.0% | Level 2: 46.9%,Level 3: 45.3%,Level 4: 7.8% | Level 2: 24.2%,Level 3: 70.1%,Level 4: 5.7% | Level 2: 26.5%,Level 3: 66.9%,Level 4: 6.6% |
| Smoking (proportion of non-smokers,%) | 67.2 | 67.2 | 59.6 | 54.8 |
| Drinking (proportion of non-drinkers,%) | 80.6 | 60.9 | 69.4 | 63.9 |
| Microbiologically proven infection (%) | 56.7 | 60.9 | 60.5 | 53.0 |
| Chronic obstructive pulmonary disease (%) | 9.0 | 4.7 | 15.3 | 13.3 |
| Cardiovascular disease (%) | 9.0 | 1.6 | 6.4 | 5.4 |

**Table S2 Dosing regimen**

| Drug | Injection | Sequential drugs |
|---|---|---|
| Nemonoxacin | Nemonoxacin malate and sodium chloride injection (Zhejiang Medicine)<br>250ml,0.5g<br>Once a day,intravenous | Nemonoxacin malate capsules (Zhejiang Medicine)<br>0.25g/per capsule<br>Once a day,2 capsules each time |
| Moxifloxacin | Moxifloxacin hydrochloride and sodium Chloride Injection (Bayer)<br>250ml,0.4g<br>Once a day,intravenous | Moxifloxacin hydrochloride tablets (Bayer)<br>0.4g/per tablet<br>Once a day,1 capsule each time |
| Levofloxacin | Levofloxacin sodium chloride injection (Daiichi Sankyo)<br>100ml,0.5g<br>Once a day,intravenous | Levofloxacin sodium chloride tablets (Daiichi Sankyo)<br>0.4g/per tablet<br>Once a day,1 capsule each time |
| Second-line therapy | Linezolid for injection (Pfizer) 300ml: 600mg/13.7g 1.2g per day<br>Tigecycline (WyethLederleS.r.l.) 50mg 0.1g daily | |
